# Aa-Z2 triggers ROS-induced apoptosis of osteosarcoma by targeting PDK-1

**DOI:** 10.1186/s12967-022-03862-1

**Published:** 2023-01-07

**Authors:** Yixin Liu, Wenyan She, Yi Li, Miao Wang, Yin Liu, Biao Ning, Tianzi Xu, Tianhe Huang, Yongchang Wei

**Affiliations:** 1 Department of Radiation and Medical Oncology, Zhongnan Hospital of Wuhan University, No. 169 Donghu Road, Wuchang District, Wuhan, 430071 Hubei People’s Republic of China; 2 Hubei Key Laboratory of Tumor Biological Behaviors, Zhongnan Hospital of Wuhan University, No. 169 Donghu Road, Wuchang District, Wuhan, 430071 Hubei People’s Republic of China; 3 State Key Laboratory of Virology, College of Chemistry and Molecular Sciences, Wuhan University, No. 299 Bayi Road, Wuchang District, Wuhan, 430072 Hubei People’s Republic of China; 4 Department of Hematology, Zhongnan Hospital of Wuhan University, No. 169 Donghu Road, Wuchang District, Wuhan, 430071 Hubei People’s Republic of China

**Keywords:** Osteosarcoma, Arsenic, ROS, Apoptosis, PDK-1

## Abstract

**Background:**

Osteosarcoma (OS) is the most frequent cancer derived from bone, and the prognosis of OS is poor. Metabolic alterations have been previously reported to contribute to the development of OS, and arsenic compounds have been suggested to exhibit strong anti-OS effects. However, few studies have described the therapeutic efficiency of arsenic compounds by targeting metabolism in OS.

**Methods:**

Here, we presented a novel organo-arsenic compound, Aa-Z2, and its antitumour efficacy against OS both in vitro and in vivo.

**Results:**

Aa-Z2 induced OS cell apoptosis, G2/M phase arrest, and autophagy through the accumulation of reactive oxygen species (ROS). Elevated ROS functioned by promoting the mitochondrial-dependent caspase cascade and attenuating the PI3K/Akt/mTOR signalling pathway. N-acetylcysteine (NAC), a kind of ROS scavenger, could reverse the effects of Aa-Z2 treatment on 143B and HOS cells. Specifically, by targeting pyruvate dehydrogenase kinase 1 (PDK-1), Aa-Z2 induced changes in mitochondrial membrane potential and alterations in glucose metabolism to accumulate ROS. Overexpression of PDK-1 could partially desensitize OS cells to Aa-Z2 treatment. Importantly, Aa-Z2 suppressed tumour growth in our xenograft osteosarcoma model.

**Conclusion:**

The study provides new insights into the mechanism of Aa-Z2-related metabolic alterations in OS inhibition, as well as pharmacologic evidence supporting the development of metabolism-targeting therapeutics.

**Supplementary Information:**

The online version contains supplementary material available at 10.1186/s12967-022-03862-1.

## Background

Osteosarcoma (OS) is the most frequent primary malignant bone tumour with an inclination to the metaphysis of children’s and adolescents’ long bones [1]. The incidence rates of OS vary throughout the world from 2 to 5 per million, and OS tends to involve early local invasion and systemic metastasis, such as lung metastasis [2, 3]. The main therapy for osteosarcoma is neoadjuvant chemotherapy combined with surgery, which has improved the 5-year survival rate to approximately 70%. Unfortunately, OS treatment has remained at a plateau in the past 30 years due to chemotherapy resistance and pulmonary metastasis [4, 5]. Therefore, there is a pressing need for safer and more effective treatment approaches.

The Warburg effect is a well-known metabolic reprogramming process in cancer and exhibits several features, including mitochondrial dysfunction and aerobic glycolysis [6–8]. As an essential glycolytic enzyme, pyruvate dehydrogenase kinase 1 (PDK-1) promotes the Warburg effect in tumor cells and reduces cell damage caused by the accumulation of reactive oxygen species (ROS). PDK-1 facilitates the metabolic switch from glucose oxidation to glycolysis in multiple cancer cells by phosphorylating the pyruvate dehydrogenase (PDH, a gate-keeping mitochondrial enzyme that converts cytosolic pyruvate to mitochondrial acetyl-CoA for oxidation) E1α subunit to inactive the PDH complex, which converts pyruvate to acetyl-coenzyme A to participate in the tricarboxylic acid cycle [9, 10]. Mounting evidence has indicated that PDK-1 is dysregulated in some malignancies and is associated with tumour proliferation, metastasis and poor prognosis [11–13]. More importantly, targeting PDK-1 provided an attractive opportunity to develop osteosarcoma treatments, and a series of novel inhibitors have been designed and synthesized to inhibit osteosarcoma growth [14–16].

Since Chinese scholars first applied arsenic trioxide (As2O3, ATO) to acute promyelocytic leukaemia (APL) treatment in the 1970s and achieved impressive efficacy, the antitumour function of arsenic compounds has been widely demonstrated in various tumours including osteosarcoma [17–20]. Arsenic exposure leads to the generation and accumulation of intracellular ROS, which can trigger a series of stress responses and cell death modes, including oxidative stress, apoptosis, autophagy, pyroptosis and ferroptosis [21–26], some of which are related to mitochondrial function and energy metabolism. In clinical work, the application of ATO (the most widely studied antitumour inorganic arsenic compound) in solid tumour treatment has limited progress because it causes strong adverse reactions [27, 28]. Therefore, in this study, we synthesized a novel organic arsenical Aa-Z2 that tended to be less toxic than inorganic arsenic with the aim of targeting cancer metabolism and discovered that Aa-Z2 could suppress osteosarcoma growth by inhibiting PDK-1.

## Materials and methods

### Materials

The following antibodies were used: Ki-67 (Cat# Ab16667) from Abcam (Cambridge, Bratian); GAPDH (Cat# 10494-1-AP), phospho-PI3K (Cat# 310163), AKT (Cat# 10176-2-AP), Phospho-AKT (Cat# 66444-1-Ig), Bax (Cat# 50599-2-Ig), Bcl-2 (Cat# 12789-1-AP), and LC3 (Cat# 14600-1-AP) from Proteintech (USA); mTOR (Cat# 380411), phospho-mTOR (Cat# 385033), SQSTM1/P62 (Cat# 380612), PDK-1 (Cat# 220521), and PDH-E1α (Cat# 385512) from ZENBIO (China); caspase 9 (Cat# 9508), cleaved-caspase 9 (Cat# 52873), caspase 3 (Cat# 9662), cleaved-caspase 3 (Cat# 9664), PARP (Cat# 9532), and cleaved-PARP (Cat# 5625) from Cell Signaling Technology (USA); PI3K(Cat# AF6241) from Affinity (USA); phospho-PDHA1(Cat# Ap1022) from Abclonal (China). N-acetyl cysteine (NAC) (Cat# A7250) was purchased from Sigma (USA). As2O3 (ATO, Cat# H20080664) was purchased from Beijing SL Pharmaceutical Company (China).

### Synthesis and characterization of Aa-Z2

The arsenic-containing compound was synthesized in two steps. First, the pro-drug 4-(1,3,2-dithiarsinan-2-yl) aniline (Z2) was described in detail. p-arsanilic acid (5 g, 18.45 mmol) and 70% ammonium thioglycolate (10 mL, 13 g, 120 mmol) were added into a 25 mL round flask containing a magnetic stir bar at 50 ℃. After 4 h, 1, 3-propanedithiol (4 mL, 23 mmol) was added dropwise and then stirred overnight to complete the reaction. Dichloromethane (DCM) and anhydrous Na2SO4 were used for extraction and drying respectively. The organic layer was purified by column chromatography on silica gel (PE/DCM = 1/4, v/v) to afford Z2 in 50% yield.

We further synthesized Aa-Z2 according to the following procedure: trimethylamine (0.25 mL, 2 mmol) was added to a solution of Z2 (0.274 g, 1 mmol) in dichloromethane (30 mL) under a N2 atmosphere. After half an hour, acryloyl chloride was added dropwise at 0 °C and stirred for 2 h. Then the reaction was quenched with H2O and extracted with CH2Cl2 (3 × 10 mL). The organic layer was washed with brine, dried over anhydrous Na2SO4, concentrated under reduced pressure and purified by column chromatography (PE/DCM = 1/50, v/v) to generate a white solid (C12H14AsNOS2, Aa-Z2) in 56% yield. In addition, Aa-Z2 was further characterized by full-scan mass spectrometry (MS) and 1H and 13C NMR spectroscopy (Additional file 1: Fig. S1).

### Cell and cell culture

Human OS cell lines (143B, HOS, MG63, U2OS) were obtained from the American Type Culture Collection (Manassas, USA). UCMSCs were purchased from Yinfeng Dingcheng Biological Engineering (Wuhan, China), AML-12 cells were purchased from Procell Life Science and Technology (Wuhan, China), and 293 T and MC3T3-E1 cells were generously gifted to us from Professor Lin Cai (Wuhan University, China). 143B and HOS cells were cultured in MEM medium (HyClone, USA) with 10% fetal bovine serum (FBS) (Gibco, USA). U2OS cells were cultured in Mycco’5A medium (Procell, China) with 10% FBS. MG63, AML12, 293 T and MC3T3-E1 cells were cultured in high-glucose Dulbecco's modified Eagle's medium (HyClone, USA) with 10% FBS. UCMSCs were cultured in RPMI-1640 medium (HyClone, USA) with 20% FBS. All cells were cultured with 100 μg/ml penicillin and streptomycin at 37 °C under an atmosphere of 5% CO2.

### Cell viability assay

All cells were plated in 96-well culture plates for 24 h at a density of 5 × 10^3^ cells/well. Then, they were treated with different doses of Aa-Z2 (0–5 μM; dissolved in DMSO, diluted in MEM medium) or ATO (0–10 μM; dissolved in saline, diluted in MEM medium) for 24 h. Cell viability was measured using the Cell Counting Kit-8 assay (CCK8, Cat# MA0218-T, Meilunbio, China). Specifically, 100 μL of medium containing 10 μL of CCK-8 dye was added to each well, and the plate was placed in a 37 °C incubator. After 2 h, the absorbance values were read at 450 nm using a microplate reader (SpectraMax M2, Molecular Devices, USA). The half maximal inhibitory concentration (IC50) values of Aa-Z2 and ATO were calculated using GraphPad Prism 8. 0 software (GraphPad, USA).

### Clone formation assay

143Band HOS cells were seeded in 6-well culture plates at a density of 1 × 10^6^ cells/well and treated with Aa-Z2 (0, 0.4, 0.6, 0.8 μM) for 24 h. After being washed three times with PBS to remove dead cells, the remaining cells were recounted and seeded in 6-well plates at a density of 500 cells/well to grow for 10–14 days. When obvious colonies were observed under an inverted microscope (Olympus, Japan), the medium was discarded, and the cells were fixed with 4% paraformaldehyde and stained with 0.1% crystal violet for 15 min. After digital photos were taken of each well, the number of colonies (> 50 cells) in each image was counted by using ImageJ software (National Institutes of Health, USA).

### Cellular ROS assay

143B and HOS cells were seeded in 6-well culture plates at a density of 1 × 10^6^ cells/well and treated with different drug concentrations (Control, Aa-Z2 0.4 μM, Aa-Z2 0.6 μM, Aa-Z2 0.8 μM, NAC 5 mM + Aa-Z2 0.8 μM, NAC 5 mM) for 24 h. Subsequently, the cells were collected and incubated with the fluorescent probe 2, 7-dichlorodihydrofluorescindiacetate (DCFH-DA, Cat#BC01010, Bioss, China). Due to the presence of background fluorescence, cells transfected with the PDK-1 overexpression plasmid were incubated with fluorescent probe dihydroethidium (DHE, Cat#C1300-2, Applygen, China). After 30 min of incubation at 37 ℃ in the dark, the cells were washed three times in serum-free medium and detected by Cytoflex flow cytometry (Beckman, USA). According to the excitation/emission wavelengths of DCFH-DA and DHE probes in the manufacturer’s instructions (488 nm/522 nm, 488 nm/610 nm), appropriate channels were selected to measure fluorescence values, and the data were analysed using Flowjo V10 software (FlowJo LLC, USA). During flow detection, the acquisition voltage, rate and gating settings were consistent for each group of cells.

### Cell apoptosis and cell cycle assays

Cell apoptosis assay was performed using an Annexin V-APC/7-AAD apoptosis kit (Cat# 70-AP105-100, Multi Sciences Biotech, China). The cell cycle was analysed by a Cell Cycle Staining Kit (Cat# CCS012, Multi Sciences Biotech, China). The cells (1 × 10^6^/well) were seeded in 6-well plates overnight and treated with different drug concentrations (Control, Aa-Z2 0.4 μM, Aa-Z2 0.6 μM, Aa-Z2 0.8 μM NAC 5 mM + Aa-Z2 0.8 μM, NAC 5 mM) for 24 h. At the indicated time, cells were collected and washed by cooling PBS three times. For apoptosis detection, these cells were resuspended in binding buffer containing 5 μL Annexin V-APC and 10 μL 7-AAD (7-amino-actinomycin D7) and plated in the dark for 10 min at room temperature. For cell cycle analysis, 1 mL DNA staining solution and 10 μL propidium iodide (PI) were added to the above cells and incubated for 30 min at room temperature. All samples were measured by Cytoflex flow cytometry at a slow flow rate (10 μL/min) and the data were analysed using CytExpert 2. 4 software (Beckman, USA). During flow detection, the acquisition voltage, rate and gating settings were consistent for each group of cells.

### Mitochondrial membrane potential assays

Mitochondrial membrane potential (MMP) was measured using an MMP assay kit with JC-1 probe (Beyotime, Cat# C2006, China). After being cultured with Aa-Z2 (0, 0.4, 0.6, 0.8 μM) for 24 h, the 143B and HOS cells were collected and stained with JC-1 dye for 20 min at 37 °C. According to the product instructions, JC-1 can aggregate in the matrix of mitochondria to form polymers with red fluorescence (488 nm/590 nm) at high mitochondrial membrane potential, while existing as monomers with green fluorescence (488 nm/529 nm). The changes in MMP were detected through Cytoflex flow cytometry and mitochondrial depolarization was measured by the percentages of JC-1 monomers. During flow detection, the acquisition voltage, rate and gating settings were consistent for each group of cells.

### ATP, lactic acid and pyruvate measurements

ATP levels were quantified by using an Enhanced ATP Assay Kit (Cat# S0027, Beyotime, China). After 24 h treatment with different Aa-Z2 concentrations (0, 0.4, 0.6, 0.8 μM), 143B and HOS cells were disrupted by 200 μL cell lysis buffer, then cell supernatant was collected after centrifugation at 12,000 ×*g* for 5 min. 20 μL supernatant and 100 μL ATP working solution were mixed for 10 s at room temperature, and the relative light unit (RLU) values were measured timely by a SpectraMax M2 microplate reader. In addition, cell culture media was obtained by centrifugation (400 ×*g*, 5 min) and the supernatant of culture media was subjected to lactic acid and pyruvate analysis using commercial kits from Jiancheng Bioengineering Institution according to the instruction manuals. (Cat# A081, Cat# A019-2-1, Nanjing, China).

### Transmission electron microscopy observations

The changes in cell ultrastructure caused by Aa-Z2 were visualized using transmission electron microscopy (TEM). 143B and HOS cells were prepared as described previously [29]. Briefly, the cells were fixed with 2.5% glutaraldehyde overnight and postfixed in 1% osmium tetroxide for 2 h. After being dehydrated in different concentrations of alcohol, the cell pellets were embedded in epoxy resin. Representative areas were chosen for ultrathin sectioning and examined by TEM (HT7700, Hitachi, Japan).

### Cell transfection, RNA extraction and quantitative Real-Time PCR

The negative control and PDK-1 overexpression plasmids were synthesized by Shanghai Jikai Company (China). 143B and HOS cells were transfected with plasmids using GP-Transfect-Mate (GenePharma, Suzhou, China) following the manufacturer’s protocol. After 48 h, total RNA was extracted with Trizol reagent (Vazyme, Cat# R401-01, China) based on the product, and then RNA was measured using a spectrometer (NanoDrop Technologies, USA). After cDNA synthesis, RT-qPCR was carried out with a CFX Connect Detector instrument (Bio-Rad, USA) and the relative mRNA expression levels were calculated by the 2^−ΔΔCt^ method [30]. The following primers were used:

GAPDH-F: 5′-GGAGTCCACTGGCGTCTTCA-3′

GAPDH-R: 5′-GTCATGAGTCCTTCCACGATACC-3′

PDK-1-F: 5′- GAGGAAGCAGGAAGGATCAGT-3′

PDK-1-R: 5′- GAACGGATGGTGTCCTGAGA-3′

### Western blotting

Radioimmune precipitation assay (RIPA) buffer containing PMSF and protease inhibitor cocktail at a ratio of 100:1:1 was used to lyse cells for 30 min. Then the supernatant was collected after centrifugation at 12,000 ×*g* for 10 min. The protein concentration was measured by using BCA Protein Assay Kit (Beyotime, Cat# P0012, China). In total, 20 μg of protein was separated by SDS–PAGE, transferred to polyvinylidene difluoride (PVDF) membrane (Merck, Cat# 05317, USA), and then blocked with 5% fat-free milk for 2 h at room temperature. All primary antibodies were diluted at a ratio of 1:1500 and incubated with the membrane at 4 ℃ overnight. The next day, these blots were incubated with HRP-conjugated secondary antibodies (1:4000) for 1 h at room temperature. Immunoreactive proteins were detected by an enhanced chemiluminescence kit (Cat# abs920, Abisin, China) according to the manufacturer’s instructions. Quantifications were performed using ImageJ software.

### Animal studies

Six- to eight-week-old male BALB/c nude mice (Charles River, China) were raised in a standard laboratory environment with food and water. An injection of a total of 2 × 10^6^ 143B cells suspended in 100 μl PBS was administered into the right axilla of mice (this day was marked as Day 0). At Day 9, these mice were randomly assigned to three groups: (1) control group: mice were injected intraperitoneally (i.p.) with saline vehicle; (2) Aa-Z2 (5 mg/kg) group: mice were injected i.p. with 5 mg/kg Aa-Z2 (dissolved in DMSO, diluted in saline, broken with ultrasonication) every 6 days; and (3) Aa-Z2 (10 mg/kg) group: mice were injected i.p. with 10 mg/kg Aa-Z2 every 6 days. The concentration of DMSO in mice was less than 1%. Animal weight and tumour size (volume = 0.5 × L × W^2^) were documented every 3 days. The mice were killed after four cycles of Aa-Z2 treatment. The tumours and vital organs were detached and fixed using 10% formalin for additional examination. All experiments were compliant with the National Institutes of Health Animal Use Guidelines and permitted by the Laboratory Animal Center of Zhongnan Hospital of Wuhan University (NO. ZN2021006).

### Statistical analysis

All data were presented as the mean of three independent experiments, which were routinely performed in triplicate, and analysed using unpaired *t* test or one-way ANOVA by using GraphPad Prism 8. 0 software. *P* < *0.05* was regarded as statistically significant.

## Results

### Aa-Z2 inhibited cell viability and induced G2/M phase arrest in human OS cells

**Fig. 1:**
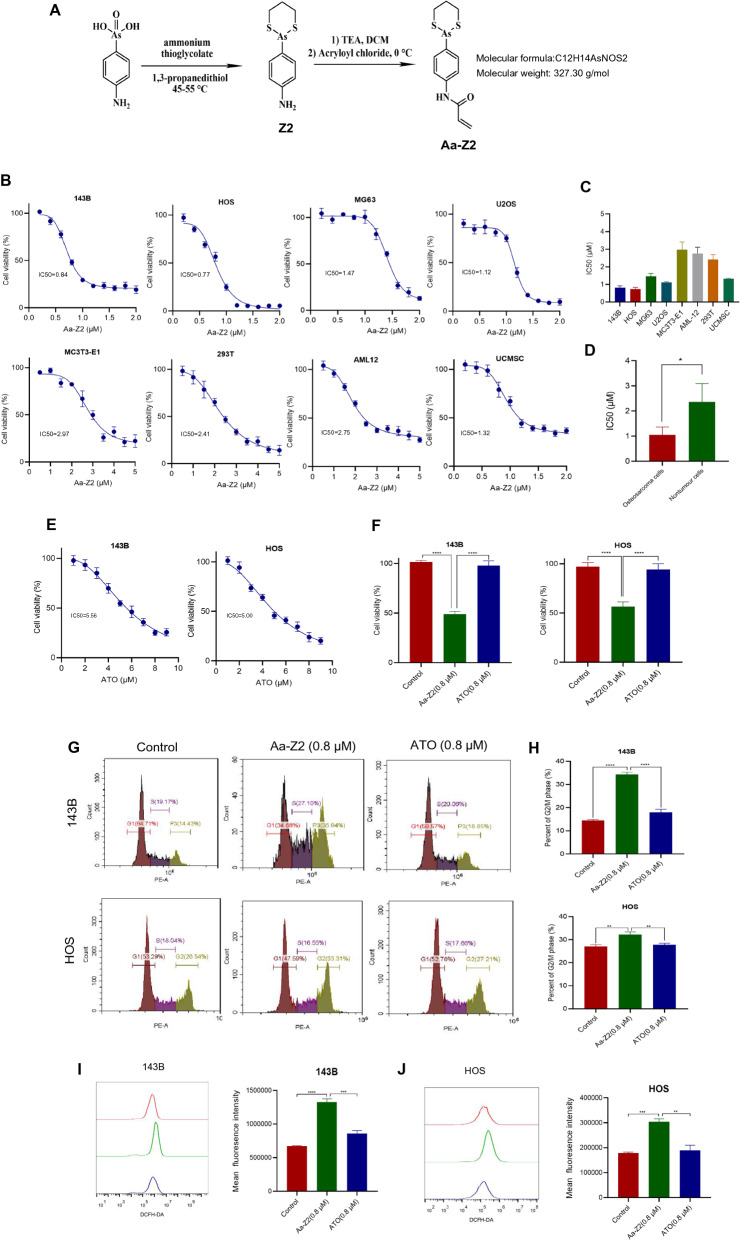
Aa-Z2 inhibited cell viability and induced G2/M phase arrest in OS cells. **A** The synthesis and structure of Aa-Z2. **B** The antiproliferative effect of Aa-Z2 on different cell lines was determined by CCK-8 assay. **C**, **D** Comparison of the IC50 values of Aa-Z2 in four OS cell lines and four nontumor cell lines. **E** The antiproliferative effect of ATO on 143B and HOS cells. **F** 143B and HOS cells were treated with Aa-Z2 and ATO for 24 h at a concentration of 0.8 μM, and cell viability was measured by CCK-8. **G**, **H** The cell cycle of 143B and HOS cells was analysed after 0.8 μM Aa-Z2 or ATO treatment for 24 h. **I**, **J** ROS levels of 143B and HOS cells were analysed after 0.8 μM Aa-Z2 or ATO treatment for 24 h

In collaboration with the College of Chemistry and Molecular Science, Wuhan University, we constructed a novel organo-arsenic compound Aa-Z2 (Fig. 1A and Additional file 1: Fig. S1). To test the toxicity of Aa-Z2, a cell viability assay was performed, and the results indicated that the cell viability of both OS cell lines (143B, HOS, MG63, U2OS) and nontumor cell lines (MC3T3-E1, 293 T, AML12, UCMSC) were markedly suppressed after treatment with Aa-Z2 (Fig. 1B). The mean IC50 values of OS cells and nontumor cells were 1.05 μM and 2.36 μM, respectively (Fig. 1C, D), indicating that Aa-Z2 was less cytotoxic towards nontumor cells than OS cells. Next, we compared the toxicity of Aa-Z2 with that of the classical arsenic compound ATO to OS cells. With a cell viability assay (Fig. 1E, F) and flow cytometry (Fig. 1G, J), we confirmed that Aa-Z2 leads to more cell viability inhibition and G2/M phase arrest in OS cells than that of ATO. Since 143B and HOS cells were more sensitive to Aa-Z2, they were selected as target cells for subsequent experiments.

### Aa-Z2 triggered cell apoptosis and cycle arrest of OS cells through ROS

**Fig. 2:**
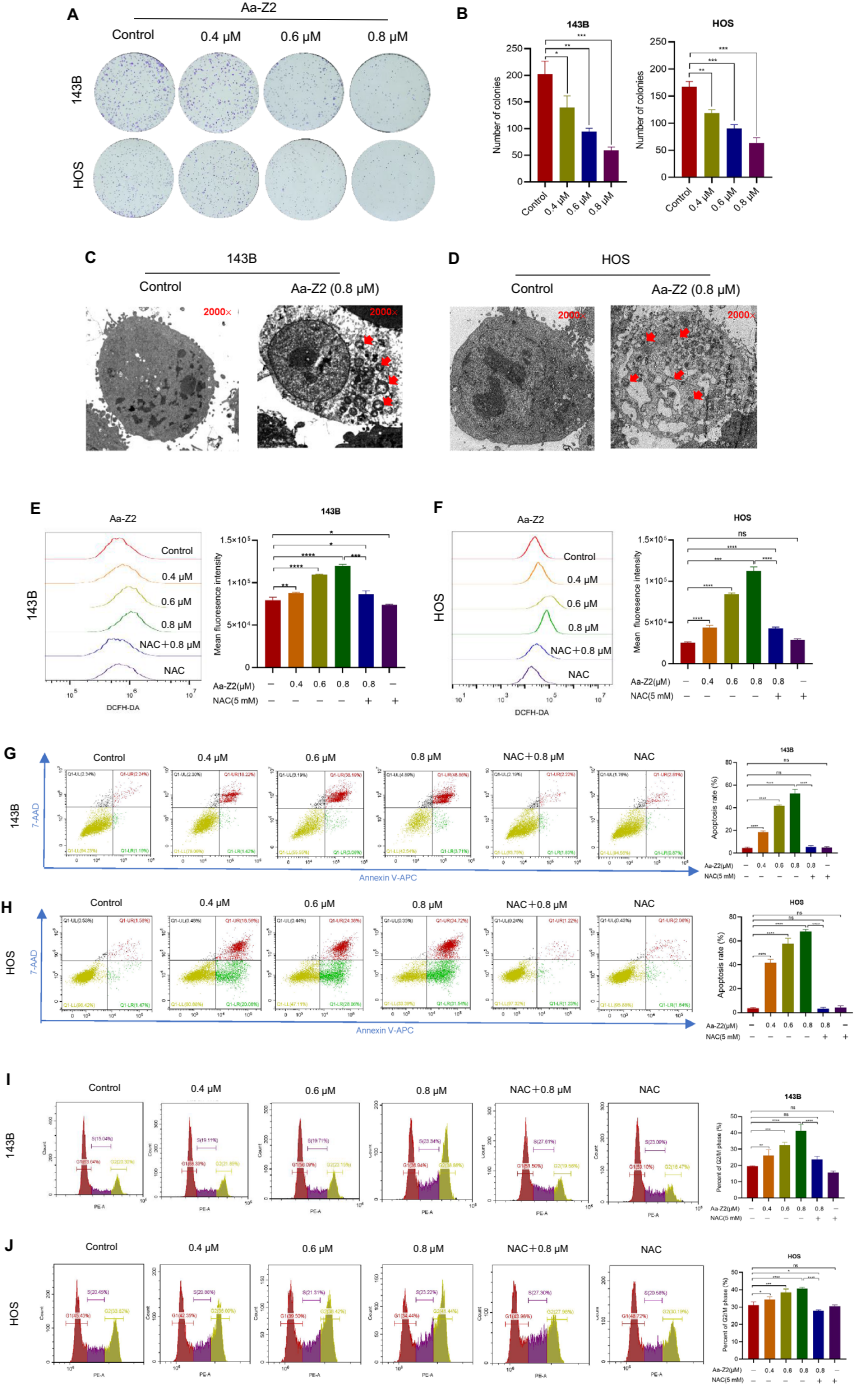
Aa-Z2 suppressed colony formation ability and induced cell apoptosis, along with increased levels of ROS in OS cells. **A**, **B** Colony formation assay for Aa-Z2-treated 143B and HOS cells. **C**, **D** Transmission electron microscopy for apoptotic bodies of 0.8 μM Aa-Z2-treated 143B and HOS cells. **E**, **F** DCFH-DA assay for ROS levels in Aa-Z2-treated 143B and HOS cells. **G**, **H** Flow cytometry detection of apoptosis when 143B and HOS cells were treated with Aa-Z2 & NAC. **I**, **J** Flow cytometry detection of G2/M phase cells when 143B and HOS cells were treated with Aa-Z2 & NAC. Mean ± SD (*P < 0.05, **P < 0.01, ***P < 0.001, ****P < 0.0001)

To investigate the anti-osteosarcoma function of Aa-Z2 in vitro, a colony formation assay was performed, and the results showed that Aa-Z2 dramatically suppressed the ability of 143B and HOS cells to form colonies in a dose-dependent manner (Fig. 2A, B). With TEM, we clearly observed obvious apoptotic bodies in 143B and HOS cells after 0.8 μM Aa-Z2 treatment (Fig. 2C, D). Since arsenic compounds usually lead to cellular ROS accumulation, we tested ROS levels in OS cells with DCFH-DA probe staining. After treatment with different doses of Aa-Z2, intracellular ROS levels increased remarkably in 143B and HOS cells. More importantly, NAC (the ROS scavenger) eliminated ROS production induced by Aa-Z2 (Fig. 2E, F). To determine whether Aa-Z2-induced growth inhibition is a result of ROS-mediated cell apoptosis and cycle arrest, OS cells were treated with different drug concentrations (Control, Aa-Z2 0.4 μM, Aa-Z2 0.6 μM, Aa-Z2 0.8 μM NAC 5 mM + Aa-Z2 0.8 μM, NAC 5 mM). As shown in Fig. 2G, H, the numbers of early apoptotic cells (Annexin V-APC positive and 7-AAD negative) and late apoptotic cells (Annexin V-APC positive and 7-AAD positive) increased significantly after treatment with Aa-Z2 at concentrations ranging from 0.4 to 0.8 μM for 24 h. Similarly, the percentage of cell populations at the G2/M phase was obviously increased after Aa-Z2 treatment (Fig. 2I, J). Moreover, these Aa-Z2-mediated changes could be rescued by NAC treatment, suggesting that Aa-Z2 could induce cell apoptosis and G2/M phase arrest through recruiting ROS in OS cells.

### Aa-Z2-induced ROS promoted the apoptosis cascade and blocked the PI3K/Akt/mTOR signalling pathway in OS

**Fig. 3:**
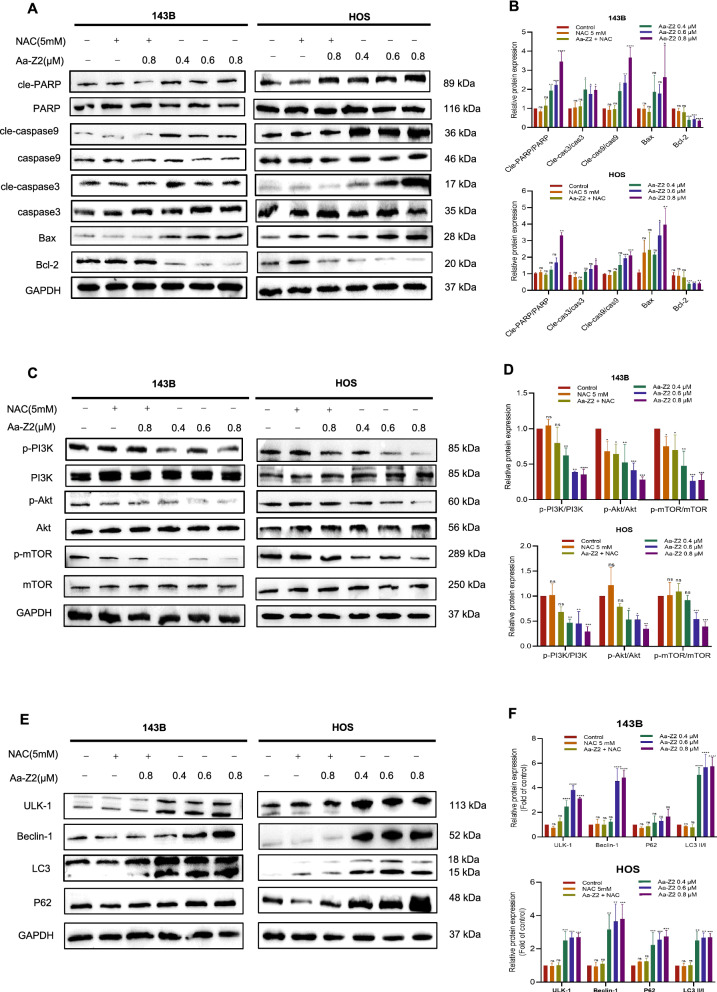
Aa-Z2-induced ROS promoted the apoptosis cascade and blocked the PI3K/Akt/mTOR signalling pathway in OS cells. **A**, **B** Apoptosis cascade detection with WB in Aa-Z2-treated 143B and HOS cells. **C**, **D** PI3K/Akt/mTOR signalling pathway assessment with WB in Aa-Z2-treated 143B and HOS cells. **E**, **F** Autophagy related pathway detection with WB in Aa-Z2-treated 143B and HOS cells. Mean ± SD (*P < 0.05, **P < 0.01, ***P < 0.001, ****P < 0.0001)

Studies have demonstrated that exposure to arsenic compounds resulted in apoptotic cascade activation and inhibition of PI3K/Akt/mTOR signalling pathway [19, 31–33]. In our study, the mitochondria-dependent apoptosis cascade (Bax/cleaved-PARP/cleaved-caspase 9/cleaved-caspase 3) was hyperactivated in both 143B and HOS cells treated with Aa-Z2 (Fig. 3A, B), and the PI3K/Akt/mTOR signalling pathway was suppressed (Fig. 3C, D), which was consistent with our observation that Aa-Z2 triggered cell apoptosis and cycle arrest in OS cells. NAC reversed the effects induced by Aa-Z2, suggesting that the changes in the apoptosis cascade and PI3K signalling pathway were ROS dependent. Another relevant pathway was autophagy activation. Specifically, Aa-Z2 upregulated LC3II/I, Beclin-1, ULK-1 and p62, while NAC restored these changes (Fig. 3E, F), which meant that Aa-Z2 strengthened autophagy was also ROS dependent. Collectively, these findings demonstrated that Aa-Z2 activated the apoptosis cascade and blocked PI3K signalling pathway through ROS.

### Aa-Z2 reprogramed glucose metabolism by targeting PDK-1 in OS

**Fig. 4:**
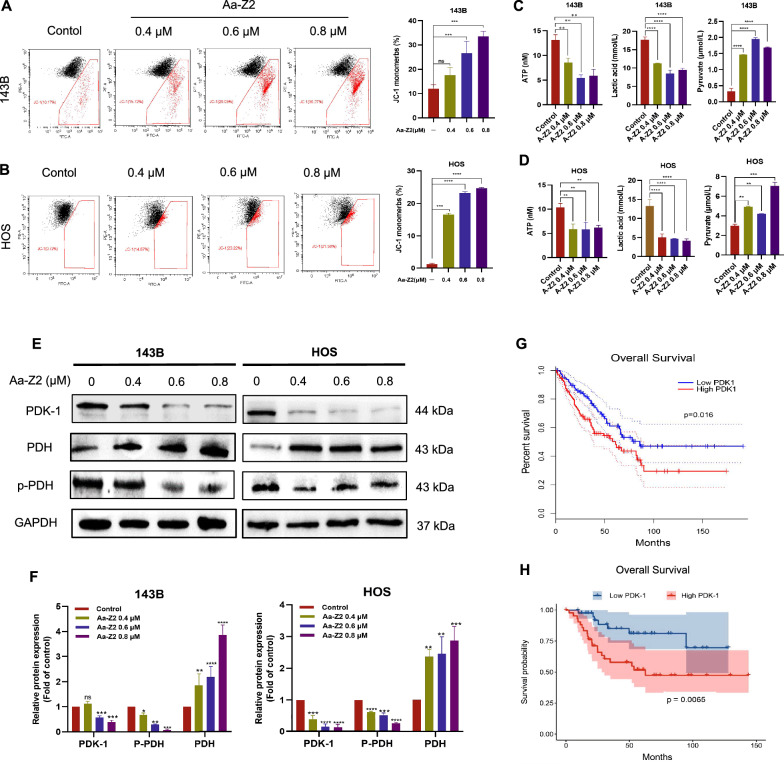
Aa-Z2 reprogramed the glucose metabolism by targeting PDK-1 in OS cells. **A**, **B** Flow cytometry analysis of JC-1 in Aa-Z2-treated 143B and HOS cells. **C**, **D** Detection of ATP, lactic acid and pyruvate in Aa-Z2-treated 143B and HOS cells. **E**, **F** Expression of PDK-1 and p-PDH was detected by WB in Aa-Z2-treated 143B and HOS cells. **G**, **H** Analysis of the relationship between overall survival and PDK-1 expression in the TCGA and TARGET datasets. Mean ± SD (*P < 0.05, **P < 0.01, ***P < 0.001, ****P < 0.0001)

Given that mitochondria are the main source of ROS [34], we hypothesized that Aa-Z2 could cause mitochondrial dysfunction to induce high ROS levels in OS cells. Then, we measured the changes in mitochondrial membrane potentials with JC-1 staining and found that Aa-Z2 sharply increased the percentage of JC-1 monomers in both 143B and HOS cells (Fig. 4A, B), indicating that Aa-Z2 drove mitochondrial depolarization. In addition, we estimated the glycolysis and glucose oxidation status in Aa-Z2-treated OS cells. As shown in Fig. 4C, D, after Aa-Z2 treatment, the generation of ATP and lactic acid significantly decreased, while pyruvate increased in 143B and HOS cells. These results indicated that Aa-Z2 reprogrammed the metabolism of OS cells. It is well-known that cell metabolism is regulated by several key enzymes, so we investigated whether Aa-Z2 targeted metabolism relevant enzymes. As discussed above, the Warburg effect is an important metabolic feature of tumour cells, and PDK-1 is a key enzyme in glycolysis, as it targets pyruvate dehydrogenase (PDH). Therefore, we detected the levels of PDK-1 and phosphorylated E1α subunit of PDH (p-PDH E1α) in Aa-Z2 treated OS cells and identified that Aa-Z2 dramatically decreased the protein expression levels of PDK-1 and p-PDH E1α (Fig. 4E, F). Together, the results indicated that Aa-Z2 suppressed PDK-1, thus shifting pyruvate metabolism from aerobic glycolysis towards glucose oxidation in OS cells. With public datasets, we confirmed the clinical relevance of PDK-1 to overall survival in OS patients. For both the TCGA and TARGET datasets, higher levels of PDK-1 were associated with worse prognosis for OS patients (Fig. 4G, H).

**Fig. 5:**
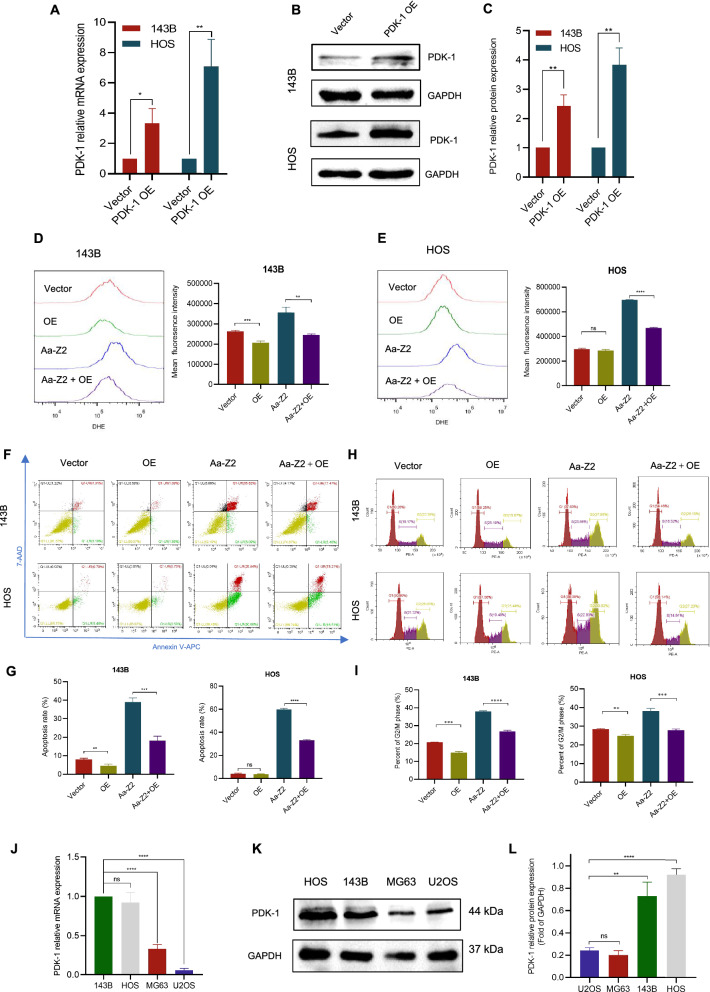
PDK-1 overexpression partially antagonized the effects of Aa-Z2 in OS cells. **A**–**C** Protein and mRNA detection in PDK-1-overexpressing 143B and HOS cells. **D**, **E** Flow cytometry detection of ROS levels in PDK-1-overexpressing & Aa-Z2-treated 143B and HOS cells. **F**, **G** Flow cytometry analysis of apoptosis in PDK-1- overexpressing and Aa-Z2-treated 143B and HOS cells. **H**, **I** Flow cytometry assessment of G2/M phase cells in PDK-1-overexpressing & Aa-Z2-treated 143B and HOS cells. **J**–**L** The expression levels of PDK-1 in four OS cell lines. Mean ± SD (*P < 0.05, **P < 0.01, ***P < 0.001, ****P < 0.0001)

To better understand the function of PDK-1 in Aa-Z2-induced cell death, we overexpressed PDK-1 in 143B and HOS cells (Fig. 5A–C), and validated that PDK-1 overexpression could partially antagonize the effects of Aa-Z2 in 143B and HOS cells by decreasing cellular ROS levels (Fig. 5D, E), apoptotic cell populations (Fig. 5F, G) and the percentages of cells in G2/M phase arrest (Fig. 5H, I). All these results suggested that PDK-1 contributed to Aa-Z2-induced OS cell apoptosis and cycle arrest. As mentioned above, compared to MG63 and U2OS cells, 143B and HOS were more sensitive to Aa-Z2. Given that PDK-1 was the target of Aa-Z2, we hypothesized that lower levels of PDK-1 in MG63 and U2OS cells led to their reduced sensitivity to Aa-Z2. With RT-qPCR and WB, we validated that the PDK-1 levels in MG63 and U2OS cells were much lower than those in 143B and HOS cells (Fig. 5J–L), which partly explained why they were less sensitive to Aa-Z2.

In conclusion, these data confirmed that Aa-Z2 triggered OS cell metabolism alteration by targeting PDK-1, contributing to accumulated ROS levels and inactivation of the PI3K/Akt/mTOR signalling pathway, as well as hyperactivation of the apoptosis cascade and autophagy.

### Aa-Z2 showed high efficiency suppressing OS growth and low toxicity in vivo

**Fig. 6:**
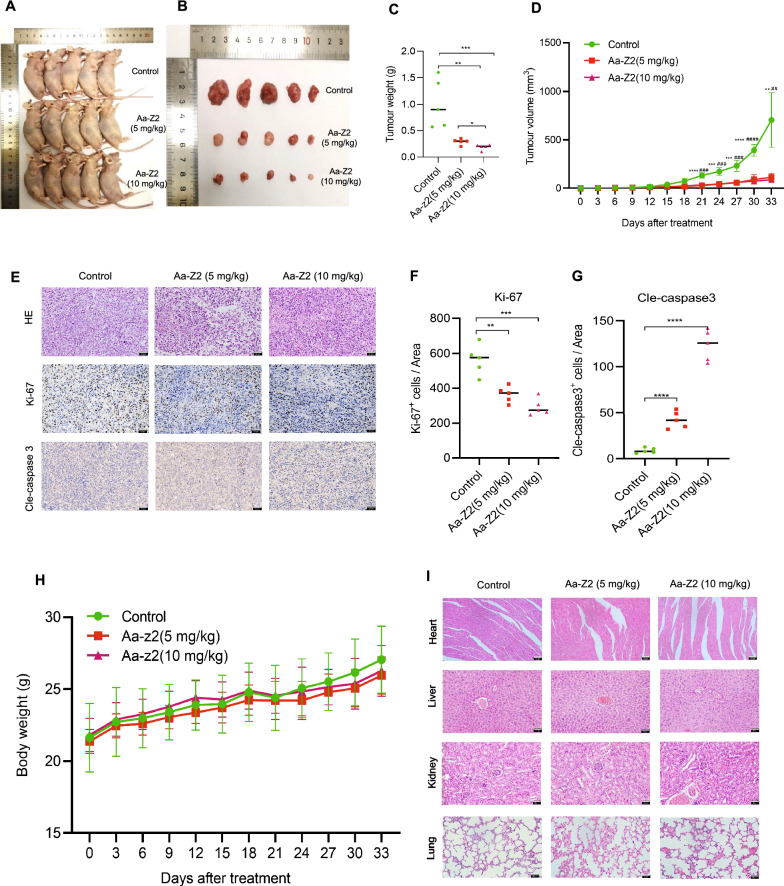
The anti-osteosarcoma effects of Aa-Z2 in vivo*.* **A**, **B** Representative images of nude mice and xenograft tumours. **C** The tumour weight of xenograft tumours. **D** Tumour volume curve of xenograft tumours. **E** Representative pictures of H&E and IHC staining for Ki-67 and Cle-caspase 3 in xenograft tumours. **F**, **G** Comparison of Ki-67- and Cle-caspase 3- positive cells among the 3 groups. **H** Mice’s body weight curve. **I** Representative images of H&E staining of the heart, liver, kidney and lung. Mean ± SD (*P < 0.05, **P < 0.01, ***P < 0.001, ****P < 0.0001)

To determine the therapeutic potential of Aa-Z2 in vivo, a cell line-derived tumour xenograft was established in male nude mice by subcutaneously injecting 143B cells. These mice were randomly divided into the control and Aa-Z2 treatment groups. All the xenografted tumours were sensitive to Aa-Z2 treatment even at a dose of 5 mg/kg (intraperitoneal injections) and received a better response at 10 mg/kg (Fig. 6A–D). IHC staining further verified that the number of Ki-67-positive cells decreased in the Aa-Z2 treatment groups, and the number of Cle-caspase3-positive stained cells in tumour tissue was significantly increased (Fig. 6E–G). These results were consistent with our observations in vitro. Then we also evaluated the safety of Aa-Z2 in mice. As shown in Fig. 6H, Aa-Z2 showed almost no effects on mouse body weight. Moreover, haematoxylin and eosin (H&E) staining of the heart, liver, kidney and lung showed that no major organ-related toxicities occurred (Fig. 6I, Additional file 1: Fig. S2). Collectively, our preclinical models supported that Aa-Z2 exhibited high efficiency and low toxicities in OS suppression.

## Discussion

Arsenic compounds represented by ATO have shown effective antitumour potential in OS [19, 24, 35, 36]. Based on previous studies, we constructed a new organic arsenic compound Aa-Z2 and demonstrated that it exhibited potent cytotoxic effects against OS both in vitro and in vivo. Aa-Z2 induces cell apoptosis, G2/M phase arrest and autophagy mediated by accumulation of ROS in 143B and HOS cells. Specifically, Aa-Z2 functioned through the ROS-triggered caspase cascade and inactivation of PI3K/Akt/mTOR signalling pathway. In vitro, we validated that ROS accumulation resulted from the Aa-Z2/PDK-1/p-PDH E1α-induced metabolism alterations and mitochondrial dysfunction.

ROS, a double-edged sword, play a critical role in regulating both the survival and death of cancer cells [37]. As byproducts of oxygen metabolism, ROS are mainly produced by mitochondrial respiratory chain, and cells maintain the balance of ROS through an antioxidant system in physiological states [38, 39]. In cancer cells, mitochondrial ROS can contribute oxidative stress to mitochondrial DNA (mtDNA), proteins and lipids, thus leading to hyperactivation of several signalling pathways from the mitochondria to the cytoplasm, such as NF-κB, MAPK, and HIF-1α, to promote the proliferation, angiogenesis and metastasis of tumours. However, irreversible cytotoxicity can be induced by high doses of superoxide through apoptosis, senescence and cell cycle arrest. In addition, enhanced mitochondrial oxidative stress results in mitochondrial dysfunction, followed by caspase activation, cytochrome C release, and cell death. According to these theories, many chemotherapeutics are used against cancer by elevating the generation of ROS [40, 41]. In our study, Aa-Z2 triggered intrinsic apoptosis mediated by ROS accumulation and showed stronger efficacy in osteosarcoma inhibition. The mitochondria-mediated pathway plays a crucial role in apoptosis because of the significant changes in mitochondrial membrane potential and related proteins, such as cleaved-PARP and caspase 3/9, in OS cells after Aa-Z2 treatment. In addition, immunohistochemical analysis revealed a substantial increase in the proportion of cell apoptosis in Aa-Z2-treated xenograft OS tissue. The imbalance of cell cycle regulation is one hallmark of cancer, and inducing cell cycle arrest may be an effective way to treat the abnormal proliferation of cancer cells [42, 43]. According to flow cytometric analysis, the proportions of OS cells in G2/M phase increased significantly after Aa-Z2 treatment, which was also reversed by NAC.

Autophagy has been widely studied because of its role in regulating cell death. During stress conditions, cytoplasmic macromolecules and organelles are seized into autophagosomes and then decomposed and digested in the lysosome [44]. Similar to ROS, autophagy provides cancer cells with protection and also causes damage accumulating studies have shown that interactions occurred between ROS and autophagy in cancer cells [19, 45]. ROS could increase the formation of autophagic flux and autophagy, in contrast, serves to restore oxidative damage. However, excessive levels of autophagy may promote cell death due by disrupting cellular homeostasis. In our study, Aa-Z2 enhanced autophagy in OS cells by ROS accumulation, which was demonstrated by a significant increase in LC3II/I, ULK-1 and Beclin-1. Surprisingly, Aa-Z2 promoted p62 expression in HOS cells. This result may be caused by the imbalance between P62 biosynthesis and degradation, which has been reported previously [45]. P62, as a substrate of lysosomal protease, is degraded during autophagy but increases under the regulation of NRF2 in a state of oxidative stress.

Malignant cancer cells tend to produce ATP through aerobic glycolysis rather than mitochondrial oxidative phosphorylation even with abundant oxygen. This is called that the Warburg effects, which helps cancer cells close the oxidative pathway and prevent the production of ROS-mediated cell apoptosis. At present, many researchers have proven PDK-1 played a crucial regulatory role in glucose metabolism because it inactivates pyruvate dehydrogenase (PDH) to prevent pyruvate from participating in the tricarboxylic acid cycle (TAC) [46, 47]. In our study, Aa-Z2 inhibited PDK-1 expression to decrease glycolysis, which was shown by the increase in PDH activity and the decrease in lactic acid. However, here, we found that long-term exposure to different concentrations of Aa-Z2 destroyed the mitochondrial function of osteosarcoma cells, resulting in pyruvate accumulation and ATP reduction. Similar results have been reported in previous studies [15]. In addition, we upregulated the expression levels of PDK-1 via plasmid transfection and found that ROS production in OS cells was partially decreased after Aa-Z2 treatment, as well as the proportions of apoptosis and G2/M phase arrest cells.

One of the limitations of our study is the potential toxicity of Aa-Z2 to normal cell lines. The toxicity of chemotherapy agents to normal tissues is a common challenge for both researchers and doctors. Our in vivo data showed that Aa-Z2 suppressed OS growth without exhibiting effects on the body weight of tumour-bearing mice (Fig. 6A–D, H). With HE staining, we also confirmed that Aa-Z2 rarely damaged important organs (Fig. 6I). To date, Aa-Z2 cannot serve as a therapeutic agent with our limited data and more studies should be performed to modulate and perfect this agent. Another limitation of our study is that 143B and HOS cells share the same patient origin [48, 49]. Since 143B and HOS cells were more sensitive to Aa-Z2, they could facilitate our functional studies on Aa-Z2, which is why the cells were selected as target cells. Collectively, more related models like osteosarcoma patient-derived xenograft model and lung metastasis model should be constructed to determine the efficacy and safety of Aa-Z2.

**Fig. 7:**
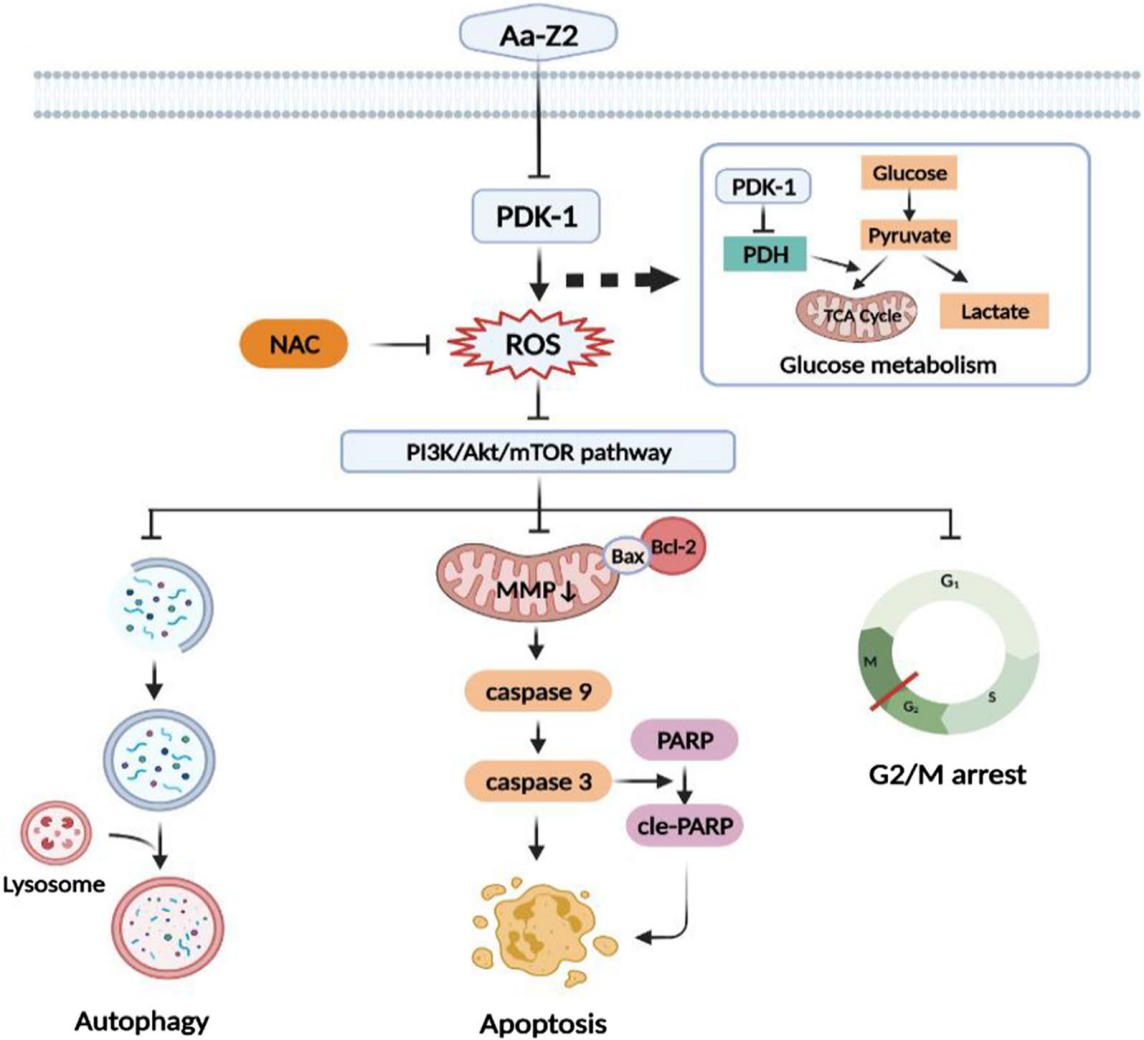
Illustration of Aa-Z2-induced metabolism reprogramming and related signalling pathway alterations in OS cells

In conclusion, we recovered a novel organic arsenic compound Aa-Z2 that reprogrammed metabolism by targeting PDK-1 and contributed to the increased levels of ROS. Accumulated ROS caused cell apoptosis, G2/M phase arrest and autophagy by triggering the mitochondria-dependent apoptosis cascade and suppressing the PI3K/Akt/mTOR pathway. Our preclinical models supported that Aa-Z2 exhibited high efficiency in suppressing OS growth and low toxicity (Fig. 7).

## Supplementary Information

**Additional file 1: Figure S1. A** The appearance of Aa-Z2 (solid, left; dissolved in DMSO, right). **B** Results of the full-scan mass spectrometry (MS). **C** Results of the 1H and 13C NMR spectroscopy. **Figure S2. A**, **B** The effects of Aa-Z2 treatment on the lungs of tumour-bearing mice.

## Data Availability

All the data involved in this study are available from the corresponding author upon reasonable request.
