# Supplementary material for: Aa-Z2 triggers ROS-induced apoptosis of osteosarcoma by targeting PDK-1
Source: J Transl Med. 2023 Jan 7;21:7. doi: 10.1186/s12967-022-03862-1 (PMC9826572; doi:10.1186/s12967-022-03862-1)
Supplement: Supplementary file 1 — Additional file 1: Figure S1. A The appearance of Aa-Z2 (solid, left; dissolved in DMSO, right). B Results of the full-scan mass spectrometry (MS). C Results of the 1H and 13C NMR spectroscopy. Figure S2. A, B The effects of Aa-Z2 treatment on the lungs of tumour-bearing mice. [file 12967_2022_3862_MOESM1_ESM.pdf]

Figure S1

A

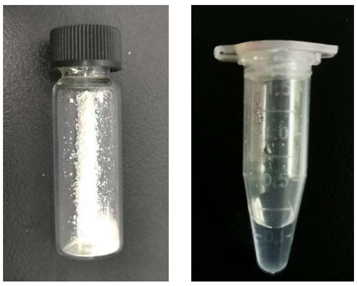

B

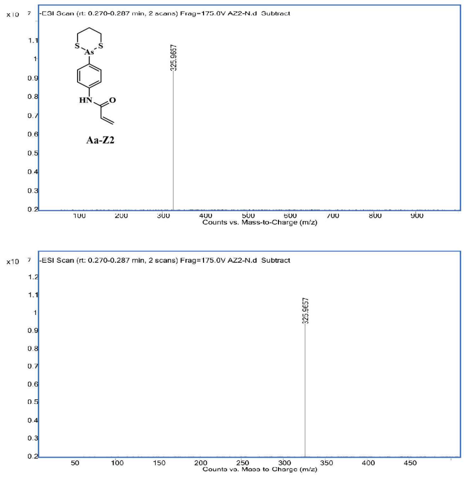

C

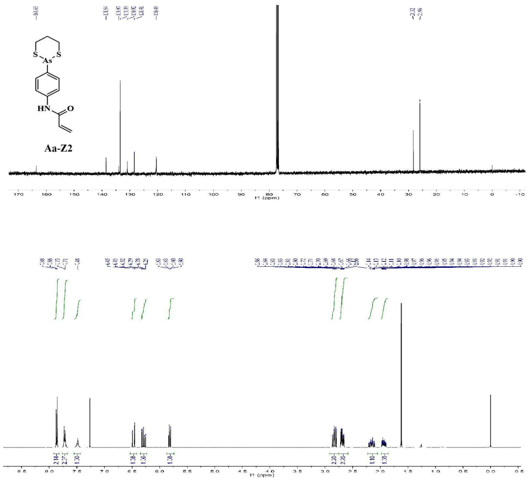

Figure S2

A

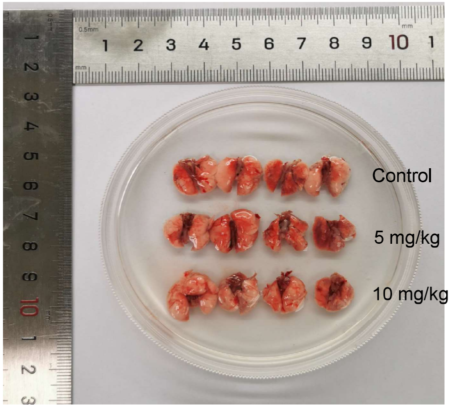

B

Control Aa-Z2 (5 mg/kg) Aa-Z2 (10 mg/kg)

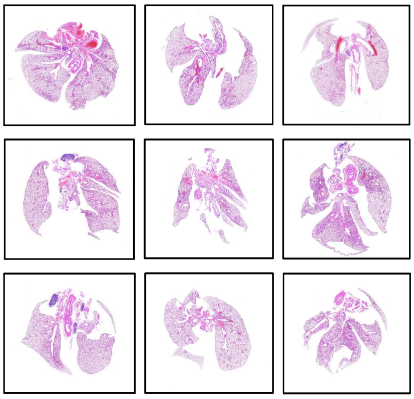

Fig S1. A The appearance of Aa-Z2 (Solid, Left; Dissolved in DMOS, Right). **B** Results of the full-scan mass spectrum (MS). **C** Results of the ^{1}H and ^{13}C NMR spectroscopy

Fig S2. A, B The effects of Aa-Z2 treatment on the lungs of tumor-bearing mice
